# Acceptability of a Healthcare Performance Evaluation System Among Professionals in Rural Areas of Ethiopia, Tanzania, and Uganda Three Years After Its Implementation

**DOI:** 10.3390/ijerph23050596

**Published:** 2026-05-01

**Authors:** Ilaria Corazza, Niyat Aregawi Gebremichael, Paolo Belardi, Fabio Manenti, Milena Vainieri

**Affiliations:** 1 Management and Health Laboratory, Sant’Anna School of Advanced Studies, Piazza Martiri della Libertà, 33, 56127 Pisa, Italy; ilaria.corazza@santannapisa.it (I.C.); milena.vainieri@santannapisa.it (M.V.); 2 Doctors with Africa CUAMM, Headquarter in Italy, Via San Francesco 126, 35121 Padua, Italy; p.belardi@cuamm.org (P.B.); f.manenti@cuamm.org (F.M.)

**Keywords:** quality in healthcare, performance evaluation, legitimacy, acceptability, qualitative method, low- and lower-middle-income countries

## Abstract

**Highlights:**

**Public health relevance—How does this work relate to a public health issue?**

- Performance measurement and evaluation are essential to improve healthcare quality, efficiency, and sustainability, particularly in low- and lower-middle-income countries (LLMICs), where health system performance gaps directly affect population health outcomes.
- The study addresses the limited legitimacy and acceptability of healthcare evaluation systems in LLMICs, a critical barrier to strengthening health systems and achieving equitable health outcomes.

**Public health significance—Why is this work significant to public health?**

- The study provides evidence that healthcare performance evaluation systems can be accepted by field professionals in LLMICs when designed appropriately, thereby supporting more effective system strengthening efforts.
- It identifies intervention coherence as a crucial factor influencing the acceptability of performance evaluation systems among local professionals, which primarily translates into their ease of understanding and use.

**Public health implications—What are the key implications or messages for practitioners, policymakers, and/or researchers in public health?**

- The findings suggest that in developing healthcare performance evaluation systems in LLMICs, experts should consider the administrative burden and opportunity cost associated with maintaining the system as factors that may hinder its acceptability.
- Policymakers and researchers should prioritize simplicity, usability, and contextual adaptation to enhance legitimacy, professional engagement, and the successful integration of evaluation systems into routine practice.

**Abstract:**

The efficacy of healthcare performance evaluation systems depends on their design and implementation, as well as on their perceived value and integration into daily practice. This study explores the acceptability of a healthcare performance evaluation system, used by health and administrative professionals in four rural healthcare settings in Ethiopia, Tanzania, and Uganda, three years after its implementation. In-depth semi-structured interviews were conducted, either in person or via video conference, with 17 professionals involved in system design and implementation. The analysis of qualitative data drew on Sekhon’s Theoretical Framework of Acceptability, using content analysis to identify themes across seven dimensions of acceptability. Key findings show that participants’ perceptions of acceptability of the performance evaluation system are influenced by data disclosure and reputational effect, the system’s understandability, alignment with their mission to improve quality of care, perceived usefulness, experienced opportunity costs, and intervention burden. The key features of the performance evaluation system are the most critical factors contributing to its acceptability, but the administrative burden, which includes professionals’ need to invest more time and change work habits to use the new system, poses some challenges and may hinder the medium- to long-term effectiveness of the intervention.

## 1. Introduction

Measuring healthcare performance is increasingly relevant, given that it is key to evaluating performance, which facilitates the achievement of health system objectives [1]. The design, development, and implementation of healthcare performance evaluation systems have emerged as a prominent topic in international health literature. Since the early 2000s, concerted efforts have been made to develop indicators tailored for monitoring, evaluating, and overseeing health systems across multiple dimensions. However, the mere design, development, and implementation of such systems are not sufficient. For these evaluation frameworks to be effective, they must also be accepted by key stakeholders. Acceptability is essential to ensure that performance measurement and evaluation systems are endorsed and embraced by relevant stakeholders, enabling them to operate effectively.

The present study explores the acceptability of a healthcare performance evaluation system (hereafter the African PES) that was developed by the Research Institute in collaboration with the Research Partner Institute since 2019 in four selected rural health districts and related reference hospitals in Ethiopia, Tanzania, and Uganda, respectively. Particularly, this article aims to investigate how the African PES has been perceived and received by health and administrative professionals in the field, three years after its development and implementation. Beyond the research scope, addressing this gap is important, as significant time, effort, and financial resources were invested in designing, developing, and implementing such system.

In a nutshell, the African PES is a voluntary governance tool supporting healthcare managers and policymakers by providing a transparent and rigorous way to measure performance. It evaluates healthcare services using 129 indicators across 8 key dimensions (i.e., local health strategies, efficiency and sustainability, users, staff and communication, emergency care, governance and quality of supply, mother and child care, and infectious and chronic diseases), covering data collected annually since 2019. Indicators are calculated at both hospital and district levels using administrative data and national health information systems. Performance is assessed through a five-level scoring system (from red to dark green), based on benchmarks and international standards where available. Results are presented using visual tools: (1) bar charts for trends and yearly evaluations, (2) a “dartboard” chart to highlight strengths and weaknesses across dimensions, and (3) a “stave” chart to show performance along patient care pathways (e.g., maternal care, infectious diseases, and chronic diseases). Overall, the PES provides a multidimensional, benchmark-based evaluation to improve healthcare quality and decision-making [2].

### Theoretical Background

Over the years, many international organizations have developed conceptual health system performance assessment (HSPA) frameworks [1,3]. These frameworks facilitate cross-country comparisons, offering standardized indicators, benchmarks, and guidelines that help countries assess performance and identify areas for improvement based on international best practices [4]. However, it is critical for countries to develop tailored frameworks and indicators that reflect their unique characteristics and priorities. Consequently, various nations have adopted different approaches [5].

High-income countries (HICs) have prioritized developing and implementing HSPAs to enhance health system performance and meet diverse population needs effectively and efficiently [6]. In contrast, low- and lower-middle-income countries (LLMICs) have made fewer efforts to establish similar systems. In fact, even when such systems exist in LLMICs, they are often applied at the national level using a top-down approach, focusing primarily on macro-level or project-level outcomes [7]. Moreover, these systems frequently target specific services or geographic areas [8,9,10] and lack comprehensive frameworks addressing the multidimensional aspects of healthcare [11,12].

To the best of our knowledge, however, a multidimensional HSPA was designed, developed, and implemented in four selected rural health districts and related reference hospitals in Ethiopia, Tanzania, and Uganda, using a bottom-up approach and with a focus on integrated healthcare—the African PES [2,13].

The African PES originated from the performance evaluation system (PES) that was initially developed in Tuscany, Italy, in 2004, by the Research Institute [14,15], and later adapted to other regional healthcare systems in Italy starting from 2008 [16] and also designed and implemented at the national level in Latvia in 2017–2019 [17]. The African PES is notable for measuring changes in health indicators over time, enabling a comprehensive analysis of annual trends related to diverse care settings at hospital and health district levels. Additionally, the framework promotes transparency by publishing annual reports, which provide accessible data for stakeholders (as an example please see the technical report in [18]). The PES is an ongoing initiative, offering opportunities to engage stakeholders in the healthcare performance evaluation process with a bottom-up approach.

Nevertheless, the design, development, and implementation of the system, as well as the regular reporting of results, on a yearly basis, are not sufficient to ensure that the system itself is used by stakeholders. Indeed, one of the most relevant issues related to performance management systems is how to engage stakeholders in the use of the provided measures and indicators.

Other scholars, for example, Otley and Ferreira [19], recognize the importance of a holistic, dynamic, and integrated approach to managing performance within organizations, with performance management systems not limited to data collection, but supportive of the use of information for the sake of decision-making, learning, and adaptation in complex environments, such as healthcare organizations. Additionally, some others, such as Broadbent and Laughlin [20], argue that organizations should adopt a context-sensitive and flexible approach to performance management rather than relying solely on rigid, top-down measurement frameworks, considering that performance measurement can create tensions between different involved stakeholders, thus requiring careful negotiation and alignment.

Hence, despite the introduction of new HSPA frameworks, the factors inherent to their use remain critical. For example, an important role is played by codesign of the systems that, among other aspects, prioritize stakeholder involvement, an iterative and adaptive process, transparency and communication, the coexistence of technical and social aspects, along with learning and capacity building [21]. Most of these peculiar traits of performance management, particularly learning, which is recognized as one of the key purposes of performance measurement [22], ensure that managing performance is most effective when organizations integrate performance data into everyday decision-making processes, rather than treating it as a bureaucratic reporting requirement [23].

Another critical factor in this use process is the system’s legitimacy, defined by Powell and Suchman [24] as “a generalized perception or assumption that the actions of an entity are desirable, proper, or appropriate within some socially constructed system of norms, values, beliefs, and definitions,” entangling at once the pragmatic, moral, and cognitive dimensions. Nonetheless, legitimacy encompasses the system’s perceived acceptability, appropriateness, and desirability within society [25]. Although the acceptability of HSPA is a critical factor as it is a key dimension of legitimacy [20,21], to the best of the authors’ knowledge, there is still no evidence up to date on the acceptability of the African PES.

## 2. Materials and Methods

### 2.1. Study Design

This study employed a qualitative research design and utilized a case study methodology [26], which is well-suited for exploring complex, real-world phenomena in their natural settings.

Specifically, the case study focused on local professionals’ experiences with the PES, with an emphasis on examining the features of PES implementation, understanding the rationale behind its adoption, exploring the strengths and weaknesses of the PES, along with prospects and opportunities for its improvement. For this reason, ethical approval requirements could be exempted, as it does not involve patients, clinical data, or experimental interventions.

All participants were informed of their rights to participate and to withdraw at any time from the study and oral consent was obtained from all those who participated in the interviews. Throughout this study, privacy and confidentiality were emphasized. All data were treated as confidential and presented only in aggregate and anonymized form. No personal identification details of any participant were linked with the information provided by them. The study was conducted in adherence to the ethical guidelines set forth in the Declaration of Helsinki.

### 2.2. Research Setting and Context

The study was conducted in four rural hospitals supported by the Research Partner Institute in Sub-Saharan Africa, in particular St. Luke—Wolisso Hospital in Ethiopia (hereafter Wolisso), Tosamaganga Regional Referral Hospital in Tanzania (hereafter Tosamaganga), along with St. Kizito—Matany Hospital (hereafter Matany) and Pope John XXIII—Aber Hospital (hereafter Aber) in Uganda. These hospitals provided a rich setting for exploring the African PES acceptability, as they also served as the context for the design, development, implementation, and use of the African PES, developed by the Research Institute, in collaboration with the Research Partner Institute, since 2019. This statement is further reinforced by the fact that, to the best of the authors’ knowledge, the African PES is the only healthcare performance evaluation system in place in LLMICs entailing at once the characteristics of multidimensionality, integration of care, timeliness, transparency, bottom-up approach, multi-stakeholder engagement, graphical representation of results, and data return.

### 2.3. Participant Recruitment

The study included health and administrative professionals from the four contexts involved in the study. In particular, local professionals that participated in the design, development, and implementation of the African PES and/or that use the system in their day-to-day work were purposely selected to provide rich insight into their experience with the PES. The recruited participants represented a diverse range of ages, genders, and professional backgrounds. Recruitment was carried out via email invitations to PES referents by hospital, or direct referrals from hospital directors. The participants willingly accepted to participate, and no signed informed consent forms were necessary before data collection began since the collected information was not sensitive.

### 2.4. Data Collection

Data were collected through in-depth interviews. Given the absence of existing instruments or literature adequately aligned with the scope of this research, a bespoke interview guide was developed for the purposes of this study. The interview guide included open-ended questions related to five different key areas, namely: (1) features of PES implementation, (2) the rationale behind PES implementation, (3) strengths and weaknesses of PES, (4) prospects and improvement opportunities of PES, and (5) concluding remarks. Open-ended questions facilitated discussion while allowing participants to narrate their experiences in their own words.

Interviews were conducted either in person or via video conferencing and lasted approximately 30 to 60 min each. With participants’ verbal consent, all interviews were audio-recorded and later transcribed verbatim. Data collection continued until data saturation was reached, at which point no new themes or insights emerged from subsequent interviews. Data saturation was determined through ongoing preliminary analysis of data already collected, which identified and confirmed that no new information or insights emerged from subsequent interviews.

### 2.5. Data Processing and Analysis

Data captured in the audio recordings were transcribed. The data were categorized and analyzed using NVivo version 12 to conduct content analysis to allow qualitative analysis of unstructured data in a coherent and logical form [27]. The analysis followed a systematic process of (1) importing transcribed interviews, (2) creating codes (tree coding) and highlighting specific portions of the text and applying these texts to the specific codes (categorization), and (3) exploring the data through various queries, applying codes and matrix queries to discern patterns and relationships within the coded content (theme development).

Transcripts were first read in their entirety to develop a holistic understanding of the data. Initial codes were then generated using a pre-determined coding framework. Codes were iteratively refined, and related codes were grouped into categories to identify overarching themes. To ensure rigor, the analysis was conducted by two researchers, who independently coded the data and reconciled discrepancies through discussion. Regular debriefing sessions and peer reviews were conducted to enhance the credibility of the findings.

The first procedural pillar underpinning the Gioia methodology [28] was applied to organize and structure the data into first- and second-order categories and then compile them into aggregate dimensions.

The analysis began with first-order coding, during which recurring words and expressions used by the participants were identified and grouped into categories. These results in first-order categories retained the participants’ original language and their specific experiences. Through open coding, each relevant piece of data was inductively classified without applying external interpretations, ensuring that the participants’ voices remained central to the analysis.

The second step involved the development of second-order themes by examining relationships among first-order categories. Through axial coding, similar or related categories were grouped, forming broader conceptual patterns.

In the final step, aggregate dimensions were generated by synthesizing the second-order themes to capture the core facilitators and barriers that influenced the decisions regarding the acceptability of the PES through the lens of the Theory of Acceptability Framework (TFA) [29].

The TFA provides a structured, evidence-based approach to evaluate the acceptability of health intervention from the perspective of people receiving or delivering it, by using seven components (or dimensions), as summarized in Table 1. The TFA has been applied in a variety of contexts (see, for example, [30,31]).

### 2.6. Ethical Approval

Ethical committee approval was not required for this study according to Italian and EU legislation on personal data protection (GDPR 2016/679 and Legislative Decree 196/2003, as amended by Legislative Decree 101/2018) [32,33,34]. The research involved voluntary interviews with participants, did not include any intervention or experimentation, and only anonymous data were collected.

## 3. Results

### 3.1. Interviews and Participants

Overall, 14 interviews were conducted between October and December 2022. A total of 18 professionals from the study settings were invited to participate in the data collection. One professional, who had initially adhered to the study, could not take part in the data collection, so a total of seventeen participants were interviewed. All interviews took place one-on-one, except four professionals (three medical records officers and one IT officer) that expressed a preference for being interviewed as a team due to logistical reasons.

Table 2 provides a comprehensive overview of the interviews conducted, while Table 3 reports selected sociodemographic characteristics of the interviewed participants.

### 3.2. Content Analysis

The findings of the content analysis are organized under the seven themes of the TFA.

#### 3.2.1. Affective Attitude

Three groups of concepts were identified when investigating participants’ feelings about the PES. The first one is the missing aspects of PES, which hinder the full success of the intervention. The second aspect reflects process feedback, especially when connected to data disclosure and reputational effects on both the hospital and professionals [35]. The last concept is related to the aim of the PES, which is to improve healthcare services. Regarding the first aspect, all interviewees emphasized their desire to broaden the implementation of the PES, aiming to encompass a larger population for a more comprehensive assessment, by highlighting the importance of benchmarking and expressing the need for the PES to be adopted in diverse countries to enable comparison:

*“But if we extend from the local level even in Ethiopia it is really good to see what the activities of the performance of the other places are, so there might be some experience sharing, we have something good that can be shared, and we have something that can be good that we can receive from other health facilities.”*

*(I_11)*

In addition to expanding the scope of the PES, participants highlighted the necessity for ongoing professional development through continuous training and learning initiatives in the future:

*“The room for improvement is about targeting the management but also going beyond the management team. Performance evaluation should involve the institution as a whole. So, to me, the greatest opportunity for improvement is to involve a larger team, rather than the management that is only the starting point. There are many more workers to be involved. Staff should be trained.”*

*(I_5)*

Furthermore, all interviewees shared their willingness to participate in future surveys, specifically tailored to healthcare workers, by briefly highlighting that such a survey could play a pivotal role in altering professional attitudes within the health sector. Accordingly, almost all professionals agreed on the integration of data found from the survey with PES:

*“I would say that a survey here could be very useful for us to get to know each other and because I see a disconnect between the data giving a direction and them not really having an idea about the indications coming from the data with the intention of changing their attitude.”*

*(I_13)*

*“[…] if we integrate this [PES] is great hand for us to utilize our data and to use the data for the future to forecast what will happen, so it is a great experience internationally for our local community. So, it will help us.”*

*(I_11)*

The second aspect identified is the acknowledgment of good results benefiting both the hospital and individual professionals. In the case of the hospital, the acknowledgment is related to a shift in its status, facilitated by the utilization of benchmarking and a commitment to data disclosure:

*“We aim at changing the status of our hospital so that our services will be recognized as specialized services, and the PES can help us in this [changing status of the hospital].”*

*(I_5)*

For individual healthcare professionals, the PES impact is revealed in the improvement of their work environment. The results have served as a validation of their efforts, fostering a sense of professional achievement and recognition:

*“[PES] is very important because you need to assess and evaluate yourself. For example, I am doing orthopedic surgeries and I need to evaluate myself every month, how many cases I did, if the patients paid for what I did, what are the outcomes of my surgeries, if the patients come back with complications so that I can check.”*

*(I_7)*

The third aspect identified is the core aim of PES, which is the continuous improvement of services through supervision and robust feedback [36]. The professionals interviewed stated the pivotal role of the PES in facilitating a process of continuous improvement within the healthcare setting. This improvement is achieved through systematic supervision that ensures adherence to standards and practices, coupled with the provision of robust feedback mechanisms. The PES, according to the respondents, serves as a valuable tool for learning from experiences [36]:

*“If it is a monitoring, you might observe or give feedback and give a corrective action since you are doing that currently. But the evaluation is retrospective, so it is learning from what has been happening.”*

*(I_12)*

*“I am personally not afraid of being evaluated because I see it as a possible way to improve the services I provide to patients; therefore, I do not perceive any stress related to being evaluated.”*

*(I_1)*

#### 3.2.2. Burden

The main emerging challenge revolves around the administrative burden [37], which entails the costs borne by individuals when engaging with the PES. Herd and Moynihan [37] describe three forms of costs: learning costs, compliance costs, and psychological costs. Based on these costs, the interviewees stated some burdens while using the PES.

Firstly, the administration burden concerns the learning costs that the stakeholders face when initially adopting the PES. According to several respondents, the system posed a certain level of complexity at the beginning, primarily due to its novelty within their organization and its divergence from the previously used system:

*“The design of the indicators, or the system in general, is burdensome; when we started the PES it was a bit complicated.”*

*(I_2)*

Secondly, the burden is associated with the additional human resources and time needed for the PES. Several respondents emphasized that the successful integration and operation of the PES demand an increased workforce dedicated to tasks such as data collection. The PES requirements for comprehensive data gathering place a strain on existing human resources, necessitating either the reallocation of personnel to other responsibilities or the recruitment of new staff members:

*“We use only the available manpower to process the data, because my department (I and my colleague) are the only ones working on data-related activities in the hospital. So, we would need more manpower and time as well.”*

*(I_14)*

Finally, the burden associated with psychological costs was experienced by some interviewees during the data collection. A respondent shed light on instances when the necessary documentation and paperwork imposed a substantial demand, occasionally escalating to a point of being overwhelmed:

*“The paperwork is very stressful and you are sometimes prone to stress.”*

*(I_2)*

In addition to the administration costs, there is a burden related to the credibility of the data sources and the decision-making processes reliant on this data. An issue lies in the perceived lack of source credibility, creating distrust in the results derived from the data:

*“[…] one of the problems I found is the data quality.”*

*(I_11)*

#### 3.2.3. Ethics

When investigating the extent to which the PES fits the previous data assessment system, two concepts were identified. Firstly, all respondents uniformly affirmed that the PES aligns with the core values, norms, and beliefs within their organization. They highlighted that the objective of the PES is aligned with their mission, which is to enhance and improve the quality of care provided, and secondly their familiarity with other performance evaluation tools that were utilized prior to the implementation of the PES in their organization:

*“We already had this kind of experience before 2019, since we were already helping collect data at the national level to compute, in part, the indicators that we have also here. We were collecting them for the Government.”*

*(I_9)*

#### 3.2.4. Intervention Coherence

When investigating the participants’ understanding of the intervention and its functionality, two concepts were identified: the main characteristics of the PES and its perceived clarity.

Firstly, the interviewees showed understanding of the key features of the PES. Their responses effectively captured the essential attributes of the system, highlighting its fundamental elements: (1) Systematic benchmarking [14]—“*PES allows comparison between different facilities, to see how your context is performing compared to the other ones*” (I_1), or “*PES uses standard indicators that are recognized officially in the international scenario and that can be shared among different healthcare settings*” (I_1). (2) Evidence-based data collection [38]—“*PES is very useful because it helps focus on the real shortcomings of the hospital, based on real evidence and scientific data*” (I_6). (3) Timeliness [39,40]—“*PES is timely and performance evaluation results are updated periodically, also allowing data visualization in trend from one year to another*” (I_1). (4) Multidimensionality [5,41,42]—“*It is comprehensive of different pathways and performance areas, not only the clinical ones, so basically that it is a multidimensional system, useful for both clinicians and managers/administrative staff”* (I_6). (5) Transparent disclosure [15,35]—“*PES helps me improve my working areas. It shows to me how I should work and what is the direction to follow. I am also open to the fact that the performance results are publicly and transparently shared within the hospital. I think it is good. For me it is a personal challenge. I know that this system can help me get better*” (I_4).

The second theme, that is perceived clarity, revolves around stakeholders’ assessments of how easily understandable the system is. Notably, stakeholders expressed their inclination toward gaining a deeper understanding of the PES, demonstrating a readiness to acquire more knowledge about its functionalities. Their proactive approach, coupled with a stated desire for learning opportunities, indicates a keen interest in enhancing their knowledge of the PES:

*“Although I am new to the PES, I immediately got what are the areas where we should work more to improve the quality of healthcare provision. I hope I will have the time to find out how you started in Tosamaganga, how you captured those differences and came up with the results. I think that will be very good practice, because we are going nowadays in a direction that requires everybody knows the data. It would be very good for us, in the health district, to have the program that you use to evaluate indicators based on standards.”*

*(I_8)*

#### 3.2.5. Opportunity Costs

It emerged that a substantial commitment of time and effort is required for data collection, urging stakeholders to seek acknowledgment and compensation for their dedicated contributions, in particular in the form of financial incentives:

*“[…] we are sacrificing ourselves to give a service [data collection] and to do what is required from us, but when you are tired, you don’t have anything to eat at the end of the day […] at least there should be some incentives.”*

*(I_2)*

Besides the challenges and efforts associated with data collection, however, it was observed that the implementation of the PES led to an additional gain in income for the stakeholders. This positive impact suggests that the system has not only presented challenges but based on managerial decisions has also contributed to tangible benefits in financial terms:

“*What we saw in the last five months is that there was also an improvement in the income, and everybody is aware of their personal achievements.”*

*(I_5)*

#### 3.2.6. Perceived Effectiveness

The success of the intervention centers on two fundamental concepts: the usefulness of the PES and the allocation of resources within it. The first concept emphasizes the pivotal role played by the PES in delivering tangible value. Stakeholders highlighted key features of the PES that proved instrumental, particularly its ability to provide evidence-based data. This feature facilitated the identification of strengths and weaknesses in the health system, thereby enriching the decision-making process. Additionally, some of the interviewees stated that the system’s effective ability to provide evidence-based data is a quality that enhances its credibility and reliability:

*“[…] based on PES data, training us on which critical areas the hospital management should focus on. Without these data we could not internalize our strengths and where we must focus […].”*

*(I_2)*

*“To me, the PES data is useful, and I think I have also managed to change some attitudes here at the hospital. Basically, […] it [PES data] adds information to the clinical data, which I receive on a monthly basis.”*

*(I_13)*

Secondly, respondents highlighted that the allocation of extra resources, encompassing financial support, technological tools, and human capital, was instrumental in providing the necessary infrastructure for the seamless operation of the system. Concurrently, targeted training initiatives were identified as a key driver to equip professionals with the requisite skills and knowledge to navigate the system adeptly:

*“Human resources were introduced in Tosamaganga to support the implementation of the PES. A hospital advisor was hired to support the general director of the hospital.”*

*(I_6)*

#### 3.2.7. Self-Efficacy

In relation to the participants’ belief in their ability to carry out the necessary actions for involvement in the intervention, two main themes emerged: confidence in task execution and the antecedent behavior of participants in utilizing the system. The first concept delves into the participants’ self-confidence in their capability to carry out the necessary tasks to participate in the intervention effectively. Indeed, most workers were confident in using the system, as the results displayed in the PES are easy to read and visualize:

*“It is very easy to read, with staves and maps, especially for clinicians like me, who are not used to analyze, read, or working with the data […].”*

*(I_6)*

The second theme, the antecedent behavior of participants in using the system, suggests that individuals’ past experiences and familiarity with the system play a significant role in shaping their confidence levels. Some of the respondents shared their antecedents with regards to the use of the PES and how they practically used the system to improve the health system:

*“The first time it was very challenging, because we were asked to provide report for three years, from 2017 until 2019. […] I used to work day and night to provide this information [PES data]. […] Then, it continued on a yearly basis and, therefore, I became kind of relaxed.”*

*(I_14)*

**Figure 1:**
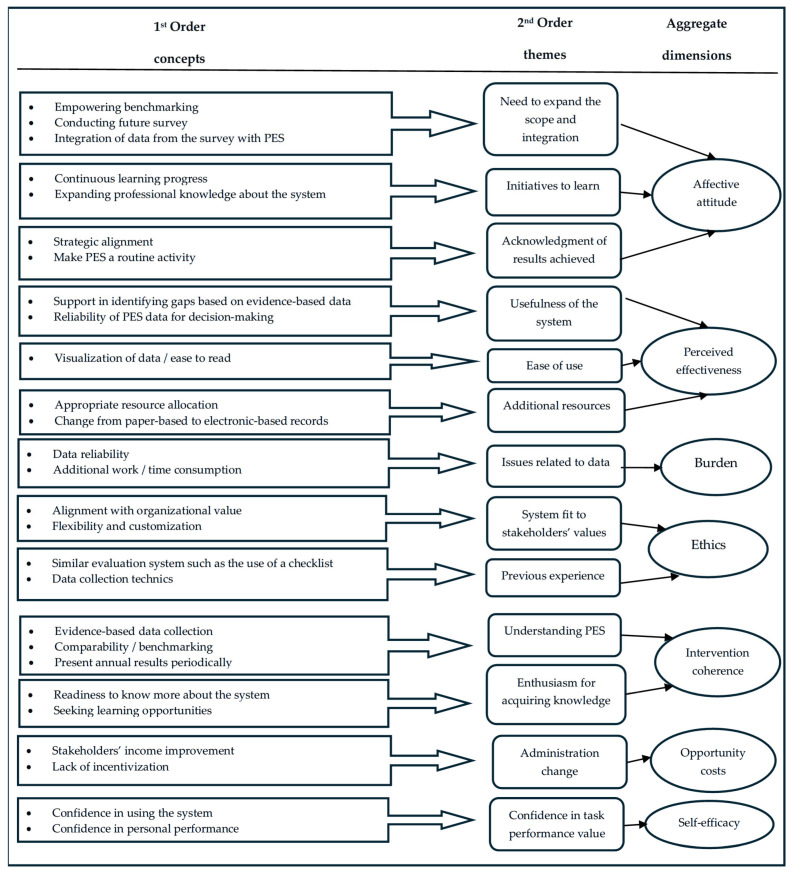
Features of PES acceptability by first- and second-order themes, and aggregate dimensions.

Figure 1 below summarizes the core characteristics of PES acceptability, based on Sekhon’s TFA, according to first- and second-order themes, and by aggregate dimension.

## 4. Discussion

It emerges from the literature that the acceptability of performance evaluation systems is seldom assessed, which might compromise the functionality of such systems despite their effectiveness. This consideration is particularly relevant in the case of LLMICs, where few assessments of the acceptability of healthcare interventions, in general, have been identified in the literature [30,31,43]. To the best of our knowledge, there is no evidence in the literature about assessing the acceptability of healthcare performance evaluation systems in LLMICs, but in all identified studies that assess the acceptability of healthcare interventions, in particular innovations and technology, it emerges that acceptability is positively related to the success of such interventions.

This study, therefore, enriches the body of literature on the acceptability of healthcare interventions in LLMICs, with a specific focus on healthcare performance evaluation systems. The implementation and use of the African PES is an original case because it allows a focus on acceptability knowing that other aspects considered as antecedents were already included into its design and implementation, such as the bottom-up approach and systematic stakeholder engagement [2].

In general, what emerges from the analysis of interview results is that the intervention and the system itself, implemented over three years at hospital and health district levels, are generally accepted by the local healthcare and administrative professionals, although some criticalities remain with regards to some specific areas.

The most represented dimension within the analysis is the intervention coherence of the PES. The key features of the PES, namely, multidimensionality [5,41,42], evidence-based data collection [38], systematic benchmarking [14], timeliness [39,40], and transparent disclosure [15,35], have been strong motivators of the healthcare and administrative workers in charge of supporting the implementation of the PES.

The multidimensional feature of the system encompasses different pathways and performance indicators, spanning various dimensions rather than focusing on a singular area, with this holistic approach aligning with the multifaceted nature of healthcare [5,41,42].

Additionally, the evidence-based data direct attention toward the authentic shortcomings of the context. By prioritizing accuracy and factual representation, decision-makers can gain a clearer understanding of the genuine challenges faced by the health system [38].

Moreover, the results are published and updated regularly, and this dynamic and real-time availability of information served as a valuable resource for decision-making. The publication of results has an important effect, especially related to a reputational effect on both the healthcare settings and professionals [15,35].

In terms of self-efficacy in using the PES, what emerges from the results is that PES users appreciate the available graphical data representation tools, feeling that they help them easily and effectively interpret the results.

The affective attitude of users that showed sincere interest in the expansion of the system and the empowered use of benchmarking at national and international levels also emerged, as well as in the opportunity to conduct surveys and integrate reported data with the PES so as to enrich it with patients’ and providers’ perspectives.

Nevertheless, there are also drawbacks inherent with the intervention, first and foremost the administrative burden [37], which includes professionals’ need to invest more time and changes in work habits to use the new system, which were challenges to the health workers. This explains their expectation and demand mainly on financial incentives to compensate with the opportunity costs related to the PES maintenance on a yearly basis. These results align well with previously conducted studies exploring opportunity costs and administrative burden in both HIC and LLMIC settings (see, as possible examples, [44,45]).

The study presents some limitations in that interviews were conducted with a purposive sample quite limited in number, because the research team included only the professionals in charge of the implementation and/or use of the PES. Beside this, it is worth noticing that the uneven number of participants across hospitals, including one case with a single interviewee, might be a source of bias; however, such effect might be partially compensated by the fact that participants were purposively selected based on their direct involvement and expertise, and that the analysis aimed to identify cross-cutting themes rather than hospital-level comparisons. If the number of professionals using and contributing to the maintenance of the PES should be increased, it would be worth checking if the results of the perception of its acceptability vary. Moreover, the interviews were conducted in English since all participants declared their full confidence in being interviewed in English, but all of them had different native languages and, therefore, the interviews conducted in native languages may have led to more refined results.

In terms of controversial aspects of this research, it may have been difficult to maintain rigor, given the significant volume of data that made analysis and interpretation time consuming, and especially the researcher’s presence during data collection, which considering the professional relationship between the research team and the study participants, may have somehow biased the responses.

Another key issue is that, as above-mentioned, the study setting is quite unique: on the one hand, because of the peculiar characteristic of the African PES that cannot be found at once in any other performance evaluation system in place in LLMICs, and on the other one because acceptability was explored among the stakeholders directly involved in the design, development, and implementation of the system itself. As such, these characteristics make the perfect combination for a case study but also make the generalizability and transferability of results to other contexts quite difficult. It is necessary that more studies are performed in this field to create a larger body of evidence that allows researchers and practitioners to generalize and transfer results across diverse research settings.

An opportunity for further research is the use of dedicated trainings—including through innovative and digital techniques—with local professionals to make the most of the African PES, checking if an increase in skills and competencies amongst a broader group of hospital and health district professionals may potentially lead to improved acceptability of the system, and reduced perception of drawbacks and burden. It would also be important to have the data collected by an external actor, who is not directly involved with the African PES, to make sure that data are collected without bias, and possibly supporting the data collection with translation and interpretation services, to overcome the limits that might emerge from dialoguing with stakeholders in English.

Theoretical implications

This study contributes to the literature in several ways. First, it extends the application of the TFA to the domain of healthcare performance evaluation systems, an area that has received limited attention compared to clinical or technological interventions. By doing so, it demonstrates the relevance of TFA dimensions, particularly intervention coherence, burden, and perceived effectiveness, in explaining the adoption and use of performance management tools.

Second, the study provides empirical evidence from LLMIC settings, where research on health system performance assessment has predominantly focused on system design rather than on stakeholder acceptance. The findings highlight that acceptability is not a secondary outcome but a core condition for the effective functioning of performance evaluation systems, thus contributing to the broader debate on legitimacy and use of performance information.

Practical implications

From a practical standpoint, the findings offer actionable insights for policymakers, healthcare managers, and system designers. First, ensuring simplicity, clarity, and usability is essential to foster intervention coherence and facilitate uptake among professionals with diverse backgrounds. Second, investment in training and capacity building is crucial to strengthen users’ confidence and maximize the benefits of performance data.

At the same time, the study highlights the need to carefully manage administrative burden and opportunity costs, which may otherwise hinder long-term engagement. This includes optimizing data collection processes, ensuring adequate human resources, and considering incentive mechanisms where appropriate.

Finally, the importance of stakeholder engagement and codesign emerges as a key factor in enhancing acceptability and embedding performance evaluation systems into routine practice. Systems that are perceived as aligned with local values and professional goals are more likely to be sustained and effectively used for decision-making and quality improvement.

## 5. Conclusions

This study set out to assess the acceptability of a multidimensional healthcare performance evaluation system implemented in rural settings in Ethiopia, Tanzania, and Uganda, three years after its introduction. The findings demonstrate that the African PES is generally well accepted by healthcare and administrative professionals, thereby confirming that performance evaluation systems can be effectively integrated into routine practice in LLMIC settings when appropriately designed.

Beyond confirming acceptability, this study contributes to the literature by identifying the key mechanisms underpinning it. In particular, intervention coherence, reflected in the system’s clarity, usability, and intuitive graphical representation, emerges as a central determinant of acceptance. This finding reinforces and extends existing evidence on the importance of usability and understanding in the adoption of health system interventions. At the same time, the study highlights that administrative burden and opportunity costs remain critical challenges, potentially undermining the long-term sustainability and effective use of such systems.

By focusing on a bottom-up, multidimensional performance evaluation system, this research provides novel empirical evidence in a field where acceptability has been largely overlooked, especially in LLMIC contexts. It shows that legitimacy is not only a matter of system design, but also of how the system is experienced in daily practice by frontline professionals.

Overall, the study demonstrates that healthcare performance evaluation systems can play a meaningful role in strengthening health systems in resource-constrained settings, provided that their design balances analytical robustness with usability and feasibility. Future research should further explore how scaling such systems and expanding stakeholder involvement may influence acceptability over time and across different contexts.

## Figures and Tables

**Table 1:** TFA construct.

Construct	Definition
Affective attitude	How an individual feels about the intervention
Burden	Perceived amount of effort required to participate in the intervention
Ethics	How well the intervention aligns with an individual’s set of values
Intervention coherence	The degree to which the participant understands the intervention and its functioning
Opportunity costs	The extent to which one needs to give up benefits, profits, or values to participate in the intervention
Perceived effectiveness	How likely the intervention is perceived to accomplish its intended purpose
Self-efficacy	The participant’s belief in their ability to carry out the necessary behaviors for engagement in the intervention

**Table 2:** Overview of the interviews conducted.

Hospital/Health District	Interview Code	Professional Profile of the Interviewee	Date of Interview	Interview Mode	Interview Format
Matany/Napak	I_1	Director of Clinical Services and Director of the Maternity Department	7 December 2022	Online	One-on-one
	I_2	Medical Records Officers (3) and IT Officer (1)	22 December 2022	Online	Group discussion (4 participants)
Aber/Oyam	I_3	Project Manager	20 December 2022	Online	One-on-one
Tosamaganga/Iringa	I_4	Director of Administration and Human Resources	24 October 2022	In person	One-on-one
	I_5	Director General	27 October 2022	In person	One-on-one
	I_6	Director of Neonatology and Pediatric Department	1 November 2022	In person	One-on-one
	I_7	Vice Director of Nursing Services	26 October 2022	In person	One-on-one
	I_8	Health District Pharmacist	31 October 2022	In person	One-on-one
	I_9	Director of Clinical Services and Director of the Maternity Department	24 October 2022	In person	One-on-one
Wolisso/Wolisso Catchment Area	I_10	Operational Manager (Wolisso Catchment Area)	5 November 2022	In person	One-on-one
	I_11	Coordinator of the Quality Unit in Wolisso Hospital	4 November 2022	In person	One-on-one
	I_12	Program Manager	4 November 2022	In person	One-on-one
	I_13	Director of Clinical Services	5 November 2022	In person	One-on-one
	I_14	IT Officer	7 November 2022	In person	One-on-one

**Table 3:** Sociodemographic characteristics of participants.

Interview Code	Gender	Age	Background	Years of Experience	
				Total Experience in the Setting Analyzed	Covering the Role in the Setting Analyzed
I_1	M	42	Gynecologist, medical doctor	14 years	8 years
I_2	3 M, 1 F	27–34	Informatics	1–3 years	1–3 years
I_3	M	65	Public health specialist, sports doctor, medical doctor	15 years	3 years
I_4	M	35	Management and human resources	1 year	6 months
I_5	M	47	Orthopedic surgeon, medical doctor	8 months	5 months
I_6	F	31	Neonatology and pediatrics, medical doctor	11 months	11 months
I_7	F	40	Nurse	7 years	2 years
I_8	M	40	Pharmacist	7 years	1 year
I_9	M	42	Gynecologist, medical doctor	14 years	3 years
I_10	M	42	Public health specialist	2 years	6 months
I_11	M	33	Health officer (specialized in reproductive and child health)	7 years	2 years
I_12	M	42	Environmental health specialist	5 years	2 years
I_13	M	66	General surgeon, medical doctor	2 years	2 years
I_14	M	37	Informatics	10 years	10 years

## Data Availability

Any data or materials related to this study are available upon reasonable request.

## Abbreviations

HIC	High-Income Country
HSPA	Health System Performance Assessment
LLMICs	Low- and Lower-Middle Income Countries
PES	Performance Evaluation System
TFA	Theory of Framework Acceptability
